# Editorial: Towards Exascale Solutions for Big Data Computing

**DOI:** 10.3389/fdata.2022.838097

**Published:** 2022-02-11

**Authors:** Domenico Talia, Paolo Trunfio, Jesus Carretero, Javier Garcia-Blas

**Affiliations:** ^1^DIMES, University of Calabria, Rende, Italy; ^2^Department of Computer Science and Engineering, Universidad Carlos III de Madrid de Madrid, Leganés, Spain

**Keywords:** big data, high performance computing, exascale systems, machine learning, parallel programming

The design and implementation of Big Data management and analysis solutions has received many benefits and improvements via the utilization of high-performance computing (HPC) systems. Today, complex processing and analysis of real-world massive data sources in AI, machine learning, and large simulations require using HPC infrastructures such as highly parallel clusters, supercomputers, and clouds (Talia, 2019). However, as parallel research and technologies advance, in the next few years, exascale computing systems will be used for implementing scalable Big Data analysis solutions in science and business (Reed and Dongarra, 2015). To reach this goal, new design and implementation challenges must be addressed and solved for exploiting the computation power of new HPC systems in running Big Data and machine learning applications.

Exascale supercomputers refer to computing systems capable of at least one exaflop or a quintillion calculations per second (10^18^). Despite their future contribution to support very large and very complex applications, exascale systems are becoming harder and harder to use efficiently (Talia et al., 2020). In particular, in the area of Big Data analysis new solutions are needed to achieve scalable software systems running quickly on exascale platforms. Extreme data refers to massive amounts of Big Data that must be queried, communicated, and analyzed in (near) real-time by using a very large number of memory and computing elements. Large repositories and continuous streams of data soon will be processed and analyzed by Exascale computing systems that today are under development (Gropp and Snir, 2013). Significant examples are scientific data produced at a rate of hundreds of gigabits-per-second that must be stored, filtered, and analyzed; millions of images per day that must be analyzed in parallel; or billions of social data posts queried in real-time on an in-memory components database. Nowadays, traditional disks and commercial storage systems cannot handle the extreme scale of data required for such applications and a very large number of cores are needed to process them. Following the need for improvement of current concepts and technologies, this Research Topic aims at focusing on data-intensive algorithms, systems, and applications running on systems composed of up to millions of computing elements on which are based the exascale systems.

Key scientific fields discussed in the papers that have been selected for this Research Topic include:

- Studies of parallel hardware and software systems for Big Data storing, processing, and analysis.
- Methods, techniques, and prototypes designed and used to implement Big Data solutions on massive HPC and exascale systems.
- Massively parallel algorithms and applications for machine learning solutions.
- New programming paradigms, APIs, runtime tools, and methodologies for expressing data-intensive tasks on exascale systems.
- Innovative applications of Big Data computing.
- Big Data analysis use cases in large-scale parallel systems.

In particular, the Research Topic includes four papers. In the paper titled “*HPTMT Parallel Operators for High-Performance Data Science & Data Engineering*,” Fox et al. introduce and illustrate the HPTMT architecture that has been developed for creating rich data applications that link all aspects of data engineering and data science together efficiently. The paper discusses an architecture using an end-to-end application with deep learning and data engineering parts working together.

In the paper “*BigFiRSt: A Software Program Using Big Data Technique for Mining Simple Sequence Repeats From Large-Scale Sequencing Data*” the authors present a Hadoop-based software system, termed BigFiRSt, to analyze Simple Sequence Repeats (SSRs) of nucleotide sequences using cutting-edge big data technology (Chen et al.). BigFiRSt consists of two major modules, BigFLASH and BigPERF, to address the problem of merging short read pairs and mining SSRs in the big data manner, respectively. Comprehensive benchmarking experiments show that BigFiRSt can reduce the execution times of fast read pairs merging and SSRs mining from very large-scale DNA sequence data.

In the paper titled “*The Old and the New: Can Physics-Informed Deep-Learning Replace Traditional Linear Solvers?*” Markidis discusses Physics-Informed Neural Networks (PINNs) that are neural networks encoding the problem governing equations, such as partial differential equations (PDE), as a part of the neural network. Physics-Informed Neural Networks have emerged as a new tool to solve challenging problems like computing linear systems arising from PDEs. The paper focuses first on evaluating the potential of PINNs as linear solvers in the case of the Poisson equation, and it characterizes PINN linear solvers in terms of accuracy and performance under different network configurations (depth, activation functions, input data set distribution).

Finally, the contribution by Kimovski et al. focuses on the “*Autotuning of Exascale Applications With Anomalies Detection*.” Autotuning automates the process of identification of the most desirable application implementation in terms of code variations and run-time parameters. The complexity and size of exascale systems make autotuning very difficult, especially considering the number of parameter variations that have to be identified. The authors introduce a novel approach for autotuning of exascale applications based on a genetic multi-objective optimization algorithm, integrated within the ASPIDE exascale computing framework. The approach considers multi-dimensional search space with support for pluggable objective functions, including execution time and energy requirements, and a machine-learning-based event detection approach capable of detecting events and anomalies during application execution, such as hardware failures or communication bottlenecks.

Those research contributions provide novel insights and solutions for the exploitation of massive parallelism in processing very large repositories of data. They describe methods and mechanisms for fostering high performance and efficiency and for offering powerful operations and tools in processing extreme data sources at high speed and/or in real-time on highly parallel computing systems, according to the high-performance data analytics (HPDA) approach.

## Author Contributions

All authors listed have made a substantial, direct, and intellectual contribution to the work and approved it for publication.

## Funding

This work was supported by the ASPIDE Project funded by the European Union's Horizon 2020 Research and Innovation Programme under grant agreement No 801091.

## Conflict of Interest

The authors declare that the research was conducted in the absence of any commercial or financial relationships that could be construed as a potential conflict of interest.

## Publisher's Note

All claims expressed in this article are solely those of the authors and do not necessarily represent those of their affiliated organizations, or those of the publisher, the editors and the reviewers. Any product that may be evaluated in this article, or claim that may be made by its manufacturer, is not guaranteed or endorsed by the publisher.
